# Application of the Updated Movement Disorder Society Criteria for Prodromal Parkinson's Disease to a Population‐Based 10‐Year Study

**DOI:** 10.1002/mds.28570

**Published:** 2021-03-17

**Authors:** Kathrin Marini, Klaus Seppi, Lena Tschiderer, Stefan Kiechl, Heike Stockner, Peter Willeit, Johann Willeit, Atbin Djamshidian, Gregorio Rungger, Werner Poewe, Philipp Mahlknecht

**Affiliations:** ^1^ Department of Neurology Innsbruck Medical University Innsbruck Austria; ^2^ VASCage, Research Centre on Vascular Ageing and Stroke Innsbruck Austria; ^3^ Department of Public Health and Primary Care University of Cambridge Cambridge United Kingdom; ^4^ Department of Neurology Hospital of Bruneck Bruneck Italy

**Keywords:** prodromal Parkinson's disease (PD), preclinical/prediagnostic Parkinson's disease, risk factors, epidemiology

In 2015, a Task Force of the Movement Disorder Society (MDS) proposed evidence‐based, probabilistic research diagnostic criteria for prodromal Parkinson's disease (PD).^1^ Studies that applied the criteria to existing longitudinal population‐based cohorts, including our own evaluation in the Bruneck Study, consistently report high specificity and negative predictive values (NPV).^2^, ^3^, ^4^ However, sensitivity and positive predictive values (PPV) varied substantially dependent on the type of study population (enriched risk vs. population‐based), depth of marker assessment, and length of follow‐up time. In 2019, a first update of the criteria was presented,^5^ which incorporated new evidence for risk and prodromal markers. It adapted likelihood ratios (LRs) for markers already included and supplemented them with four new markers.

We have now applied the updated criteria to the Bruneck Study cohort and assessed differences in original versus updated probabilities for prodromal PD within and across groups. Detailed information on the study population, design, and assessments including the application of the original criteria for prodromal PD was published previously^3^, ^6^ and is additionally outlined in the Appendix. In brief, 539 participants without PD or secondary parkinsonism at baseline in 2005 (55–94 years; 290 females) were reassessed after 5.0 (range: 4.9–5.0) and 10.4 (10.4–10.5) years for incident PD.^3^ Baseline probabilities were modeled retrospectively including 16 of the 18 risk and prodromal markers originally included by the MDS Task Force.^1^ For the present updated analysis, all four newly included markers^5^ were available and integrated into the algorithm: type 2 diabetes mellitus (clinical diagnosis, antidiabetic medication, and/or glycated hemoglobin [HbA1c] values >6.4%), physical inactivity (according to the Baecke physical activity questionnaire), low plasma urate in men, and global cognitive deficit (Mini‐Mental State Examination [MMSE] <25/30 points).

While median baseline probabilities for prodromal PD decreased from 2.4% (25^th^–75^th^ percentile: 0.6%–8.7%) using original criteria to 1.8% (0.4%–8.1%; *P* < 0.001) with updated criteria, 12 participants met the ≥80% probability threshold for probable prodromal PD using original criteria as compared with 16 using updated criteria. A correlation analysis between original probabilities and updated probabilities is presented in upper row of Fig. 1. Updated probabilities in participants with incident PD were higher than the original ones (Fig. 1, middle row), while in participants who remained free of PD during follow‐up, updated probabilities were lower than the original ones (*P* < 0.001; Table S1). However, these significant divergent changes (Fig. 1, lower row) did not translate into higher predictive accuracies of the updated criteria for incident PD in our sample, as in absolute numbers only a few participants with incident PD were reclassified (Tables S1 and S2).

**FIG. 1.**
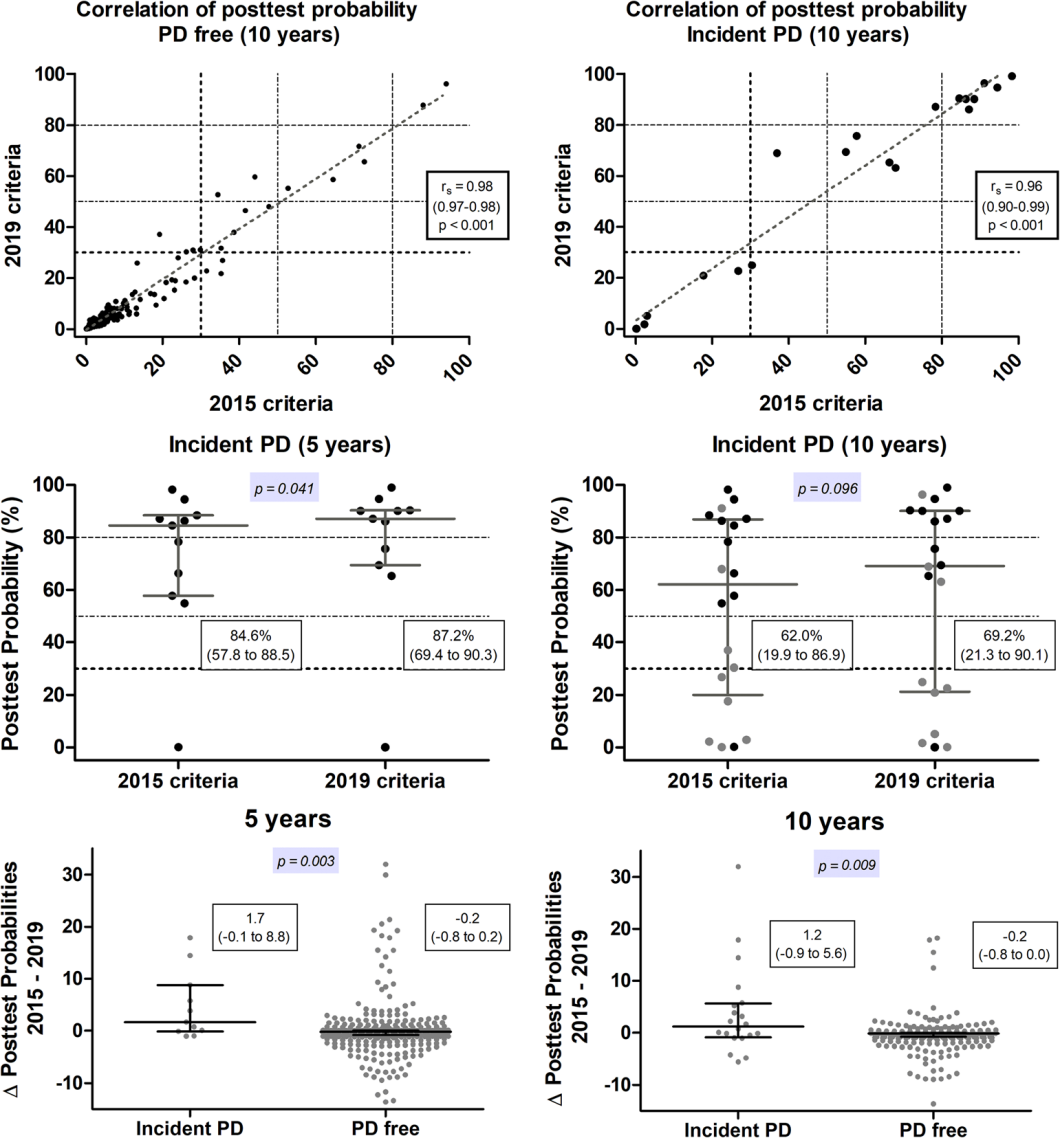
Upper row: scatterplot and Spearman rank correlation analyses with original probabilities (2015 criteria) plotted on the x‐axis and updated probabilities (2019 criteria) on the y‐axis. Numbers in parentheses indicate 95% confidence intervals. The upper dashed lines represent the 80% probability cutoff for probable prodromal Parkinson's disease (PD) as defined per Movement Disorder Society (MDS) research criteria, the middle dash‐dotted and the lower dotted lines represent 50% and 30% probability cutoffs, also mentioned by the MDS Task Force.^1^, ^7^ Middle row: scatterplots of the absolute baseline posttest probability for prodromal PD, illustrated for cumulative cases with incident PD at 5‐year and 10‐year follow‐ups. Incident cases at respective follow‐ups are shown in black and incident cases from previous follow‐ups in are shown in lighter gray. The resulting predictive accuracy measures are given in Table S2. Lower row: scatterplots of the relative change in posttest probabilities with the two criteria versions (probability according to updated criteria minus probability according to original criteria) in the various groups. In the middle and lower rows, single values are given with the respective group median (25^th^ to 75^th^ percentiles) and p‐values were calculated with the paired Wilcoxon signed‐rank test (middle row) or Mann‐Whitney U test (lower row). [Color figure can be viewed at wileyonlinelibrary.com]

In summary, we previously reported our findings of the original criteria in the unselected population‐based Bruneck Study cohort showing a moderate to high predictive accuracy in identifying cases of incident PD over up to 10 years of follow‐up.^3^ Predictive accuracy did not change when using the updated criteria, probably due to the low sample size and thus low number of converters despite the long follow‐up time. Nevertheless, the updated MDS criteria were superior to the original MDS criteria with regard to separating individuals with low and high probabilities likely indicating presence of prodromal PD. Therefore, together with one other study published to date that has applied the updated criteria,^4^ our findings speak to the MDS Task Force Bayesian classifier methodology that allows for sequential inclusion of new markers as they become available. The results of the present study should be confirmed by larger prospective population‐based studies, which should include markers with high LR.

## Author Roles

K.M.: study concept, acquisition of data, statistical analysis and interpretation of data, drafting of the manuscript, and manuscript revision.

K.S.: obtaining funding, study concept, acquisition of data, statistical analysis and interpretation of data, and manuscript revision.

L.T.: statistical analysis and interpretation of data, and manuscript revision.

S.K.: obtaining funding and interpretation of data, designing the Bruneck Study, and manuscript revision.

A.D.: acquisition of data, and manuscript revision.

H.S.: acquisition of data, and manuscript revision.

P.W.: data management, statistical analysis and interpretation of data, and manuscript revision.

J.W.: obtaining funding, designing the Bruneck Study, and manuscript revision.

G.R.: acquisition of data, and manuscript revision.

W.P.: obtaining funding, interpretation of data, and manuscript revision.

P.M.: obtaining funding, study concept, acquisition of data, statistical analysis and interpretation of data, drafting of the manuscript, and manuscript revision.

## Full Financial Disclosure for the Previous 12 Months

K.M.: reports a grant from the Tiroler Wissenschaftsförderung (Grant UNI‐0404/2245).

K.S.: reports personal fees from Teva, UCB, Lundbeck, AOP Orphan Pharmaceuticals AG, Roche, Grünenthal, Stada, Licher Pharma, Biogen, and Abbvie; honoraria from the International Parkinson and Movement Disorders Society; and research grants from FWF Austrian Science Fund, Michael J. Fox Foundation, and AOP Orphan Pharmaceuticals AG, outside the submitted work.

L.T.: nothing to report.

S.K.: reports grants from Tirol Kliniken, Tyrolean Health Insurance Company, Tyrol Health Care Funds, Boehringer, Nstim Services, Sanofi, and the Austrian Research Promotion Agency (FFG) during the conduct of the study, and personal fees from Amgen, Bayer, Boehringer, Pfizer, Medtronic, and Sanofi, and non‐financial support from Boehringer outside the submitted work.

A.D.: reports honoraria from AbbVie outside the submitted work.

H.S.: nothing to report.

P.W.: reports Support from Novartis Pharmaceuticals, Bayer, Daiichi Sankyo, and Sanofi outside the submitted work.

J.W.: nothing to report.

G.R.: nothing to report.

W.P.: reports personal fees from Alterity, AbbVie, Affiris, AstraZeneca, BIAL, Biogen, Britannia, Lilly, Lundbeck, Neuroderm, Neurocrine, Denali Pharmaceuticals, Novartis, Orion Pharma, Roche, Takeda, Teva, UCB, and Zambon (consultancy and lecture fees in relation to clinical drug development programs for PD); royalties from Thieme, Wiley Blackwell, Oxford University Press, and Cambridge University Press; and grant support from MJFF; EU FP7, and Horizon 2020 outside the submitted work.

P.M.: reports lecture fees from Boston Scientific outside the submitted work and a grant from the Tiroler Wissenschaftsförderung (Grant UNI‐0404/2245).

## Supporting information


**Appendix S1.** Supporting Information.
