# Supplementary material for: Application of the Updated Movement Disorder Society Criteria for Prodromal Parkinson's Disease to a Population‐Based 10‐Year Study
Source: Mov Disord. 2021 Mar 17;36(6):1464–6. doi: 10.1002/mds.28570 (PMC8251993; doi:10.1002/mds.28570)
Supplement: Supplementary file 1 — Appendix S1. Supporting Information. [file MDS-36-1464-s001.docx]

**SUPPLEMENTARY MATERIALS**

**Application of the updated MDS criteria for prodromal Parkinson’s disease to a population-based 10 year study**

**Supplementary methods**

**Study design and assessment of probabilities for prodromal PD according to the original criteria**

Detailed information on study design as well as ascertainment and definition of risk markers and prodromal markers for the retrospective application of the original Movement Disorders Society (MDS) criteria for prodromal Parkinson’s disease (PD)^1^ to the prospective population-based Bruneck study can be found in our previous publications.^2,3^ The protocols of the Bruneck Study had been approved by the local ethics committee and all participants had given written informed consent according to the declaration of Helsinki.

In brief, the follow-up assessment of 2005 included a variety of clinical motor and non-motor signs and risk markers relevant for PD and was used as a baseline for this analysis (574 individuals aged 55–94 years, representative for the general elderly community). Presence of clinically defined PD was ascertained per United Kingdom PD Society Brain Bank criteria. We excluded 17 participants with secondary parkinsonism and 18 participants with idiopathic PD at baseline, leaving 539 participants as the baseline population for the present analysis.

All markers included by the MDS task force^1^ that are easy and quick to assess were available for retrospectively modeling baseline probability for prodromal PD:^2,3^ male sex, pesticide exposure, solvent exposure, caffeine intake, smoking status, first-degree relative with PD, substantia nigra hyperechogenicity on transcranial sonography, possible subthreshold parkinsonism (assessed by the UPDRS-III), probable REM-sleep behavior disorder (RBD), olfactory impairment, constipation, excessive daytime somnolence, orthostatic hypotension, urinary dysfunction, severe erectile dysfunction, and depression/anxiety. Dopamine transporter activity, polysomnography confirmed RBD, and genetic testing were not available.

**Assessment of probabilities according to the updated criteria**

A first update of the MDS research criteria was presented in 2019,^4^ incorporating new evidence for risk and prodromal markers published since 2015. It adapted likelihood ratios (LR) for markers already included and supplemented them with four new markers.

Therefore, for the present updated analysis posttest probabilities for prodromal PD were calculated by (1) using adapted LRs as detailed by Heinzel el al. 2019^4^ and (2) integrating LRs for the four newly integrated markers, on which data was recorded during the general part of the Bruneck study 2005 (baseline).

New risk markers in the updated analysis included diabetes mellitus type II, lower physical activity as well as lower serum urate levels in male participants. Furthermore, as the only new prodromal marker, assessment of cognitive deficits was included in the updated analysis.^4^

Presence of diabetes mellitus type II was rated with a LR+ for a HbA_1C_ value > 6.4%, use of antidiabetic medication in participant’s premedication as well as a clinical diagnosis of diabetes mellitus type II in participant’s medical history. LR- was applied in absence of these criteria.

Physical activity was assessed with the Baecke Physical Activity Questionnaire.^5^ It evaluates physical activity of individuals over the previous 12 months. The questionnaire consists of three indices: a work index, a sport index and a leisure time index. A score of 5 indicates highest activity levels and a score of 1 indicates the least activity for each index. The total score is calculated by adding the three indices (minimum of 3, maximum of 15 points). Participants’ scores were classified into activity tertiles of high, intermedium and low physical activity.^6^ Low physical activity was rated with LR+, while high physical activity was rated with LR- (LR=1 for the intermediate tertile of activity).

For serum urate levels in male participants a cut-off of <5.0 mg/dl was rated with LR+. LR- was applied for serum urate levels >5.6 mg/dl. For male participants with a serum urate level of 5.0-5.6 mg/dl and for all female participants a LR=1 was given.^4^

Cognition was assessed with the Mini Mental State Examination. A cut-off of <25/30 points was rated with LR+, indicating cognitive decline, while a score of >26/30 points was rated with LR-. MMSE Scores of 25-26 were rated as intermediate results with LR=1.^7,8^

Generally, we chose a conservative approach by applying LR+ or LR- only if markers were unambiguously present or absent, and borderline cases were rated with a neutral LR of 1 as suggested by the MDS task force.^1,4^ Missing values were also included in the calculation with a LR of 1.

**Follow-up**

Follow-up visits took place after a median of 3.0 (range 3.0-3.4) years during an interim study in 2008 and during the regular Bruneck Study in 2010 and 2016 and allowed for identification of incident PD cases by two neurologists blinded to baseline risk markers. Due to the low number of converters after three years, we focused our analysis on 5-year and 10-year data with cumulative incident PD cases (for a detailed flowchart of the study population see Supplementary Figure 1).

**Statistical analysis**

Calculation of baseline pretest probability, LRs, and posttest probability was performed according to the updated MDS research criteria.^4^ Non-parametric tests were used to compare original and updated probabilities within and across groups (see Supplementary Table 1 legend). Receiver-operating-characteristic-based area under the curve (AUC) of probabilities for the differentiation of incident cases from those who remained PD-free were calculated along with 95% confidence intervals (95%CI). Then, sensitivity, specificity, positive predictive value (PPV), and negative predictive value (NPV) of baseline probable prodromal PD status (i.e. ≥80% post-test probability) for incident PD were calculated. We repeated calculations using lower cut-offs of 50% probability and 30% probability for prodromal PD, as originally discussed by the MDS task force as a broader definition for what might be called ‘possible prodromal PD’.^1,9^ SPSS 26.0 (IBM Corp., Armonk, NY) was used for all statistical analyses. Statistical tests were two-sided and the significance level was set to p<0.05.

**Supplementary Figure 1:** Flowchart of the Bruneck Study: participant numbers, dropout rates and diagnosis of incident PD for the respective analysis


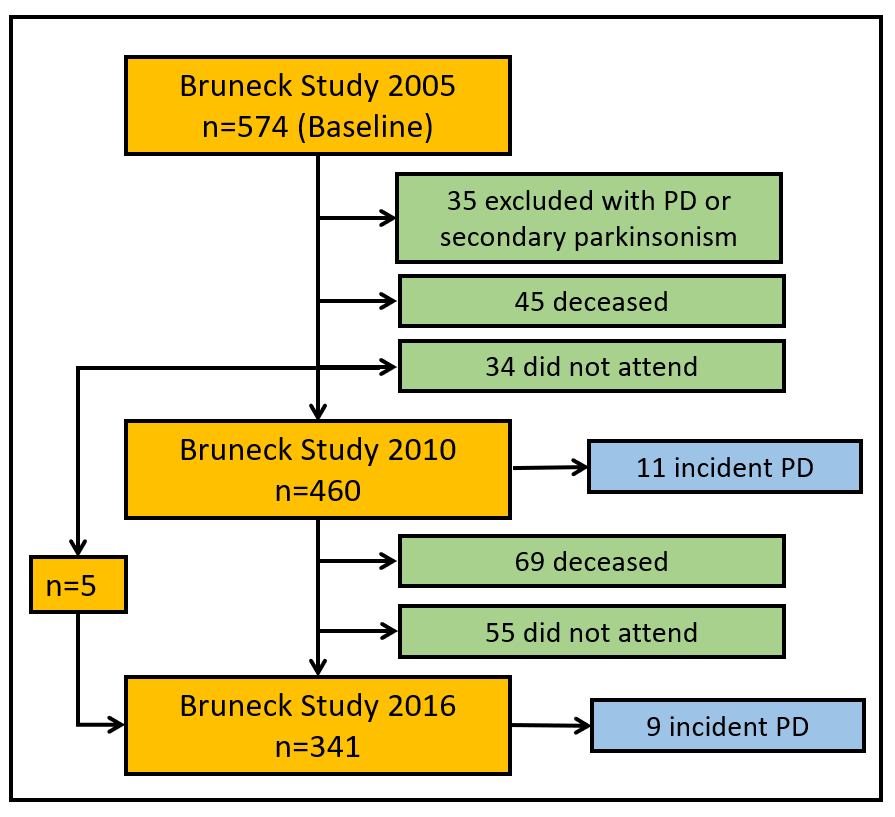


**Supplementary Table 1: Baseline probabilities according to original 2015 and updated 2019 criteria**

|  | **Cross**  **sectional**  **baseline** | **Follow-up 0–5 y**  **(n=465)** | | | | **Follow-up 0–10 y**  **(n=341)** | | | |
| --- | --- | --- | --- | --- | --- | --- | --- | --- | --- |
|  | **PD-free**  **(n=539)** | **Incident PD**  **(n=11)** ^d^ | **PD-free**  **(n=454)** | **p-**  **Value** ^e^ | **AUC Value**  **(95%CI)** | **Incident PD**  **(n=20)** ^d^ | **PD-free**  **(n=321)** | **p-**  **Value** ^e^ | **AUC Value**  **(95%CI)** |
| **Age ^a^** | 67.2  (61.4 to 76.2) | 75.6  (71.6 to 78.8) | 65.9  (60.9 to 74.0) | 0.009 |  | 70.5  (63.4 to 76.2) | 64.2  (59.6 to 69.6) | 0.004 |  |
| **M/F (n) ^b^** | 249 / 290 | 5 / 6 | 214 / 240 | 0.99 |  | 11 / 9 | 162 / 159 | 0.82 |  |
| **Posttest probability**  **(2015 criteria) ^a^** | 2.4%  (0.6 to 8.7) | 84.6%  (57.8 to 88.5) | 1.9%  (0.5 to 6.5) | <0.001 | 0.90  (0.75 to 1.0) | 62.0%  (19.9 to 86.9) | 1.2%  (0.4 to 4.6) | <0.001 | 0.86  (0.74 to 0.98) |
| **Posttest probability**  **(2019 criteria) ^a^** | 1.8%  (0.4 to 8.1) | 87.2%  (69.4 to 90.3) | 1.4%  (0.3 to 5.8) | <0.001 | 0.90  (0.74 to 1.0) | 69.2%  (21.3 to 90.1) | 1.0%  (0.2 to 3.8) | <0.001 | 0.86  (0.74 to 0.99) |
| **p-value** ^c^ | <0.001 | 0.041 | <0.001 |  |  | 0.096 | <0.001 |  |  |
| **Δ Posttest probability** | -0.2%  (-0.8 to 0.2) | 1.7%  (-0.1 to 8.8) | -0.2%  (-0.8 to 0.0) | 0.003 |  | 1.2%  (-0.9 to 5.6) | -0.2%  (-0.8 to 0.0) | 0.009 |  |
| **≥80% (2015 / 2019 criteria) ^b^** | 12 / 16 | 6 / 7 | 4 / 6 |  |  | 7 / 8 | 2 / 2 |  |  |
| **≥50% (2015 / 2019) criteria ^b^** | 30 / 36 | 10 / 10 | 13 / 17 |  |  | 12 / 13 | 6 / 8 |  |  |
| **≥30% (2015 / 2019 criteria) ^b^** | 55 / 60 | 10 / 10 | 33 / 34 |  |  | 14 / 13 | 15 / 16 |  |  |

Abbreviations: AUC = Area under the curve; LR = Likelihood ratio; PD = Parkinson’s disease; 95%CI = 95% Confidence interval

^a^ Results represent medians (25^th^ ­to 75^th^ percentile).

^b^ Results represent numbers of participants in categories.

^c^ p-values in row represent significances for differences between probabilities calculated with the original 2015 criteria versus the updated 2019 criteria within groups and were calculated with the paired Wilcoxon signed-rank test (two-sided).

^d^ Cumulative incident PD cases.

^e^ p-values in columns represent significances for differences of incident PD cases versus those who remained PD-free at respective follow-ups and were calculated with the Mann-Whitney-U test (two-sided).

**Supplementary Table 2:** Predictive accuracy of ‘probable’ or ‘possible prodromal Parkinson’s disease’ for incident Parkinson’s disease

**(A) According to original 2015 criteria** (as reported previously in Mahlknecht et al. *Mov Disord* 2018)^2^

|  | **Probability cut-off** | **Sensitivity** | **Specificity** | **PPV 1 (per protocol) ^b^** | **PPV 2 (intention to analyze) ^c^** | **NPV** |
| --- | --- | --- | --- | --- | --- | --- |
| **Follow-up 0–5 y:**  **PD-free (n=454) vs. incident PD (n=11) ^a^** | **80%** | 54.6% (28.0–78.8) | 99.1% (97.7–99.7) | 60.0% (31.2–83.3) | 50.0% (25.4–74.6) | 98.9% (97.4–99.6) |
|  | **50%** | 90.9% (60.0–99.0) | 97.1% (95.1–98.4) | 43.5% (25.6–63.2) | 33.3% (19.1–51.3) | 99.8% (98.6–99.9) |
|  | **30%** | 90.9% (60.0–99.0) | 92.7% (90.0–94.8) | 23.3% (13.0–37.9) | 17.9% (9.8–30.0) | 99.8% (98.5–99.9) |
| **Follow-up 0–10 y:**  **PD-free (n=321) vs. incident PD (n=20) ^a^** | **80%** | 35.0% (18.0–56.8) | 99.4% (97.6–99.9) | 77.8% (44.3–94.7) | 58.3% (31.9–80.7) | 96.1% (93.9–97.5) |
|  | **50%** | 60.0% (38.6–78.2) | 98.1% (95.9–99.2) | 66.7% (43.6–83.9) | 40.0% (24.6–57.7) | 97.5% (95.1–98.8) |
|  | **30%** | 70.0% (47.9–85.7) | 95.3% (92.4–97.2) | 48.3% (33.4–65.6) | 25.5% (15.7–38.4) | 98.1% (95.8–99.2) |

**(B) According to updated 2019 criteria**

|  | **Probability cut-off** | **Sensitivity** | **Specificity** | **PPV 1 (per protocol) ^b^** | **PPV 2 (intention to analyze) ^c^** | **NPV** |
| --- | --- | --- | --- | --- | --- | --- |
| **Follow-up 0–5 y:**  **PD-free (n=454) vs. incident PD (n=11) ^a^** | **80%** | 63.6% (35.2–85.0) | 98.7% (97.1–99.5) | 53.9% (29.1–76.8) | 43.8% (23.1–66.9) | 99.1% (97.7–99.7) |
|  | **50%** | 90.9% (60.0–99.0) | 96.3% (94.0–97.7) | 37.0% (21.5–55.8) | 27.8% (15.7–44.1) | 99.8% (98.6–99.9) |
|  | **30%** | 90.9% (60.0–99.0) | 92.5% (89.7–94.6) | 22.7% (12.7–37.2) | 16.7% (9.1–28.2) | 99.8% (98.5–99.9) |
| **Follow-up 0–10 y:**  **PD-free (n=321) vs. incident PD (n=20) ^a^** | **80%** | 40.0% (21.8–61.4) | 99.4% (97.6–99.9) | 80.0% (47.9–95.4) | 50.0% (28.0–72.0) | 96.4% (93.7–98.0) |
|  | **50%** | 65.0% (43.2–82.0) | 98.1% (95.9–99.2) | 61.9% (40.8–79.3) | 36.1% (22.4–52.5) | 97.5% (95.1–98.8) |
|  | **30%** | 65.0% (43.2–82.0) | 95.0% (92.0–97.0) | 44.8% (28.4–62.5) | 21.7% (13.0–33.8) | 97.8% (95.3–99.0) |

Abbreviations: NA = not applicable; NPV = negative predictive value; PD = Parkinson’s disease; PPV = positive predictive value.

^a^ Analysis based on cumulative incident PD cases.

^b^ PPV 1 based on cases with available data at respective follow-up (outcome based).

^c^ PPV 2 based on all baseline cases (more conservative).

**References**

1. Berg D, Postuma RB, Adler CH, et al. MDS research criteria for prodromal Parkinson’s disease. Mov. Disord. 2015;30(12):1600–1611.

2. Mahlknecht P, Gasperi A, Djamshidian A, et al. Performance of the Movement Disorders Society criteria for prodromal Parkinson’s disease: A population-based 10-year study. Mov Disord. 2018;33(3):405–413.

3. Mahlknecht P, Gasperi A, Willeit P, et al. Prodromal Parkinson’s disease as defined per MDS research criteria in the general elderly community. Mov Disord. 2016;31(9):1405–1408.

4. Heinzel S, Berg D, Gasser T, Chen H, Yao C, Postuma RB. Update of the MDS research criteria for prodromal Parkinson’s disease. Mov Disord. 2019;34(10):1464–1470.

5. Baecke J, Burema J, Frijters J. A short questionnaire for the measurement habitual physical activity in epidemiological. Am J Clin Nutr. 1982;36(5):936–942.

6. Voorrips L, Ravelli A, Dongelmans P, Deurenberg P, Van Staveren W. A physical activity questionnaire for the elderly. Med Sci Sport Exerc. 1991;23(8):974–979.

7. Kvitting AS, Fällman K, Wressle E, Marcusson J. Age-Normative MMSE Data for Older Persons Aged 85 to 93 in a Longitudinal Swedish Cohort. J Am Geriatr Soc. 2019;67(3):534–538.

8. Creavin ST, Wisniewski S, Noel-Storr AH, et al. Mini-Mental State Examination (MMSE) for the detection of dementia in clinically unevaluated people aged 65 and over in community and primary care populations. Cochrane Database Syst Rev. 2016 Jan 13;(1):CD011145.

9. Berg D, Postuma RB, Bloem B, et al. Time to redefine PD? Introductory statement of the MDS Task Force on the definition of Parkinson’s disease. Mov Disord. 2014;29(4):454–462.
